# Cross-sight transjugular intrahepatic portosystemic shunt (CS-TIPS): combined ultrasound-fluoroscopy guidance for simultaneous percutaneous portal and hepatic vein puncture and access closure with plug deployment

**DOI:** 10.1007/s11547-026-02175-3

**Published:** 2026-01-27

**Authors:** Maria Giovanna Riga, Sonia Triggiani, Sveva Mortellaro, Salvatore Alessio Angileri, Anna Maria Ierardi, Simone Raoul Mortellaro, Gianpaolo Carrafiello

**Affiliations:** 1 Postgraduation School in Radiodiagnostics, University of Padua, 35122 Padua, Italy; 2 Postgraduation School in Radiodiagnostics, University of Milan, Via Festa del Perdono 7, 20122 Padua, Italy; 3 Radiology Department, Fondazione IRCCS Cà Granda, Policlinico di Milano Ospedale Maggiore, 20122 Padua, Italy

**Keywords:** TIPS, Cross-sight (CS) technique, Ultrasound guidance, Portal hypertension, Interventional radiology

## Abstract

Transjugular Intrahepatic Portosystemic Shunt (TIPS) is a well-established procedure for managing portal hypertension and its complications that are refractory to medical and endoscopic therapy. However, complex cases may render the conventional approach ineffective. We describe the cross-sight (CS) technique, which combines ultrasound and fluoroscopic guidance to allow simultaneous puncture of the hepatic and portal veins through a single transhepatic needle pass. Following portal access, stent graft deployment is performed via the jugular route, while the percutaneous tract is closed with a vascular plug to minimize bleeding risk. In our experience, this approach enabled safe and effective shunt creation after failed standard TIPS, with no procedure-related complications. The CS technique provides enhanced procedural control and avoids the limitations of alternative strategies such as DIPS or gun-sight methods. By reducing puncture attempts and access points, it represents a feasible salvage option for complex TIPS cases, with potential immediate application in interventional radiology practice.

## Introduction

Transjugular Intrahepatic Portosystemic Shunt (TIPS) placement is a standard approach for managing portal hypertension-induced complications, including refractory ascites and variceal bleeding [1–3]. In 5–10% of cases, the standard approach fails [4, 5], so alternative access strategies have been described in select cases [6, 7]. We describe a case in which TIPS was placed via percutaneous combined sonographic and fluoroscopic guidance (cross-sight (CS) technique), enabling serial puncture of both the portal and hepatic veins with a single transhepatic needle pass. Written informed consent was obtained from the patient.

## Case presentation

A 59-year-old woman with HBV-related cirrhosis and prior partial portal vein thrombosis—on oral anticoagulation—presented with massive upper gastrointestinal bleeding from esophageal varices unresponsive to endoscopic treatment. Emergency TIPS was indicated to control portal pressure and prevent rebleeding. Pre-procedural contrast-enhanced Computed Tomography (CE-CT) scans revealed partial recanalization of the right portal vein.

## Conventional approach

Access via the right internal jugular vein with a 10 Fr sheath (Rosch–Uchida Transjugular Liver Access Set, Cook Medical). The right hepatic vein was accessed. Despite attempts under fluoroscopic (Azurion ClarityIQ, Philips Healthcare) and transhepatic ultrasound (EPIQ 5 Elite, Philips Healthcare) guidance, portal vein access failed.

## Patient preparation

Local anesthesia at the puncture site was achieved by subcutaneous injection of a solution of 10 ml of 2% lidocaine. The procedure was performed with continuous anesthesia assistance, in particular with moderate sedation of the patient, using a combination of midazolam (0.07–0.08 mg/kg), propofol (0.5–2 mg/kg), and fentanyl (1–2 lg/kg) administered i.v. Heart rate, electrocardiographic tracing, oxygen saturation, and respiratory rate were continuously monitored. Blood pressure was determined every 4 min. Antibiotic prophylaxis against infection was provided with 2 g of i.v. cefazolin sodium just before the procedure.

## Cross-sight technique step by step

**Fig. 1:**
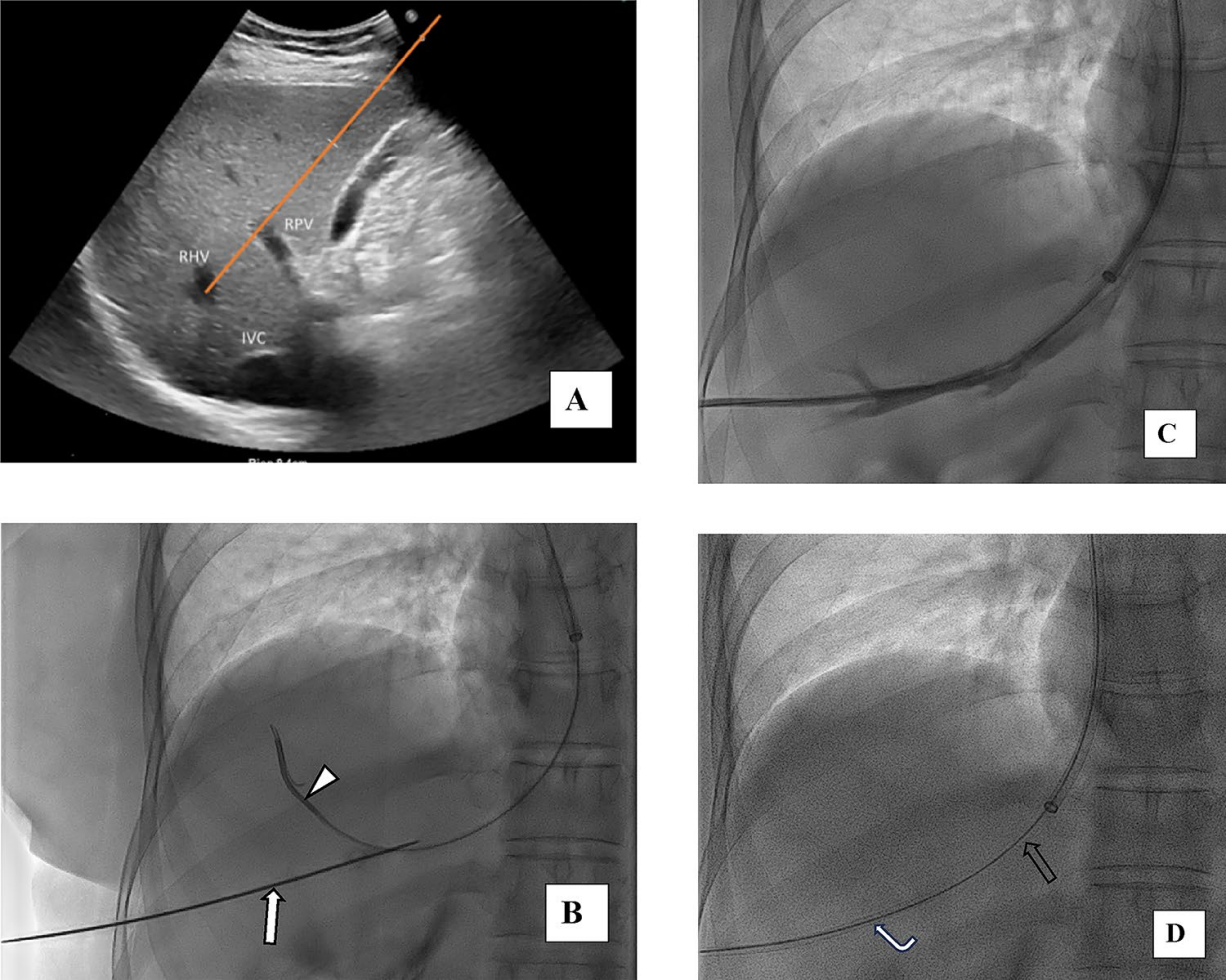
**A** Pre-procedural ultrasound demonstrating puncture of the right hepatic vein in the same plane as the portal vein; **B** Cranio-caudal tilting shows overlap of the Ring needle (arrow) introduced via the transhepatic access with the catheter (arrowhead) previously positioned in the VSE through the jugular approach; **C** Contrast injection through the transhepatic Ring needle confirming correct access to the hepatic vein; **D** advancement of the 6F introducer (curved arrow) over the Terumo Floppy wire (hollow arrow)

**Fig. 2:**
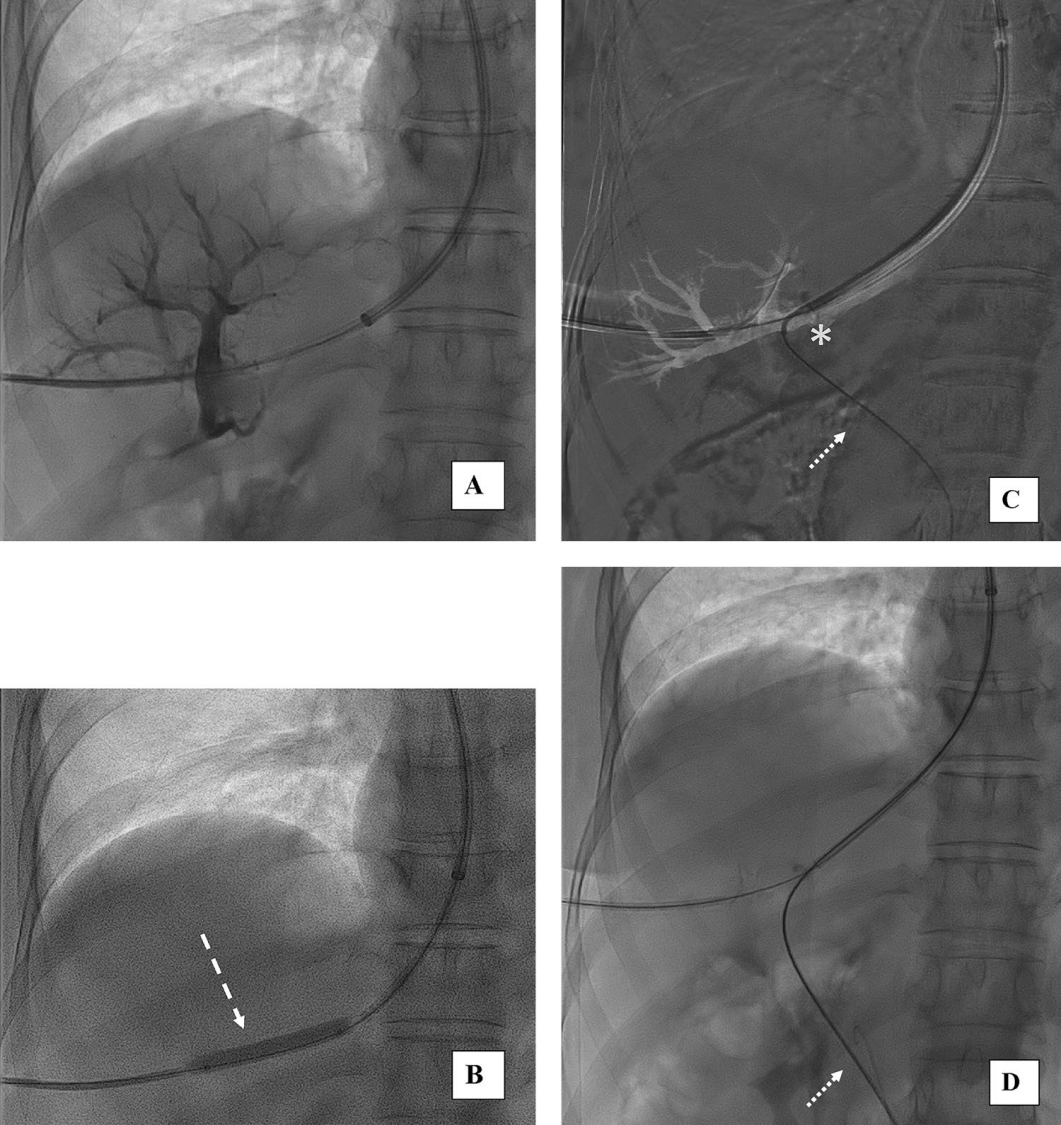
**A** fluoroscopy confirmed the correct puncture of a right portal vein branch with contrast media injection through transhepatic sheet pulled back; **B** angioplasty balloon (5 × 40 mm) (dashed arrow) dilatation of the tract between the hepatic and portal vein; **C** selective catheterization of the main portal vein using a 5 Fr vertebral catheter (asterisk) and a hydrophilic guidewire (dotted arrow) through a 65 cm 7F sheath inserted through the jugular access; **D** hydrophilic guidewire in the portal vein via jugular approach replaced by a stiff guidewire (dotted arrow)

**Fig. 3:**
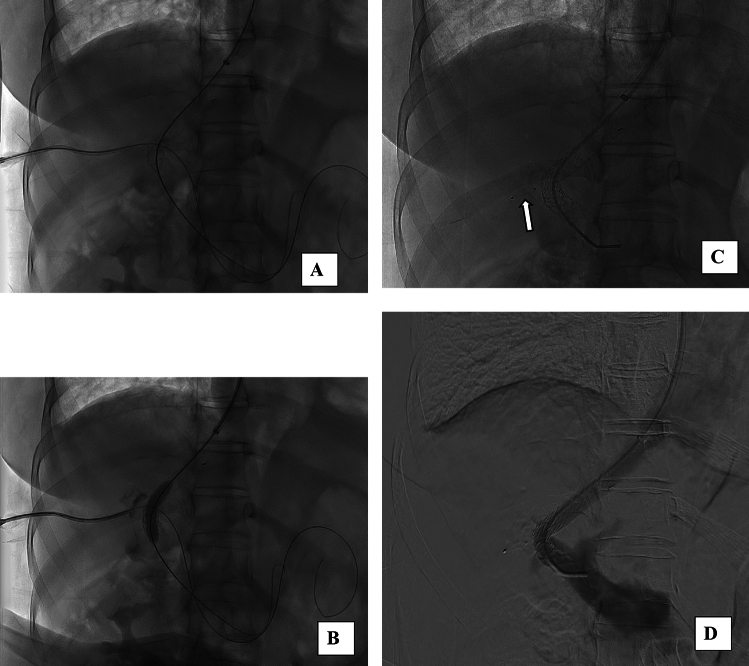
**A** Viatorr stent graft 7 × 10 mm deployed between the right portal vein and the right hepatic vein; **B** post-dilatation to 8 mm using an angioplasty balloon; **C** Amplatzer vascular plug (arrow) deployed under fluoroscopic guidance to secure the percutaneous hepatic tract; **D** confirmation of correct TIPS positioning

1. A percutaneous transhepatic sonographic serial puncture of both the right portal vein and the right hepatic vein using a single transhepatic needle pass was performed using a 5F Needle (Ring Drainage Catheter Needle Set, Cook Medical Europe Limerick, Ireland) Fig. 1A.
2. A diagnostic catheter (Vertebral 5F, 65 cm, Cordis Corporation, FL, USA) was placed in the hepatic vein from the jugular access and it was used as a target to better perform hepatic vein puncture combined with US guidance. The correct placement of the 5F needle inside the hepatic vein was confirmed with cranio-caudal tilting of C-arm which demonstrated the overlapping of the Ring needle with the catheter inserted into the right hepatic vein Fig. 1B.
3. Injection of contrast media through the 5F Needle confirmed access to the hepatic vein Fig. 1C.
4. A hydrophilic guidewire (Angled, 0.035’’, 180 cm, Terumo Europe, Leuven Belgium) was introduced through the transhepatic access site up to the right hepatic vein and then to the IVC. Transhepatic needle was exchanged with a 6-French sheath (Radiofocus Introducer, Terumo Europe, Leuven Belgium).
5. Subsequently, a 20-mm diameter Gooseneck Snare (Medtronic, Plymouth, MN, USA) was inserted via the jugular access to snare the wire inserted via transhepatic way, thereby establishing through-and-through access (flossy wire) Fig. 1D.
6. Then the 6F transhepatic sheath was pulled back up to see blood flow, and injection of contrast media confirmed the access in to the right portal vein Fig. 2A.
7. The tract between the hepatic and portal veins was then dilated using a 5 × 40 mm angioplasty balloon catheter (Mustang TM, PTA Balloon Catheter, Boston Scientific, Pulau Pinang, Malaysia) advanced over the wire from the jugular approach Fig. 2B.
8. After balloon dilatation, the main portal vein was selectively catheterized via the jugular route using a 5-French vertebral catheter and a hydrophilic guidewire through a 65 cm 7F sheath (Destination Terumo Europe, Leuven Belgium) inserted from the jugular access Fig. 2C.
9. Through the 6F transhepatic introducer, a hydrophilic guidewire was advanced into the portal vein to stabilize the transhepatic access pathway.
10. The hydrophilic guidewire advanced in portal vein via jugular approach was then exchanged with a stiff guidewire (Boston Scientific, Amplatz Super Stiff TM 0.035’’, 180 cm, Costa Rica) Fig. 2D, over which a 7 cm × 10 mm Viatorr stent graft (Gore, Flagstaff, Arizona, USA) was deployed between the right portal vein and the right hepatic vein Fig. 3A.
11. The stent was post-dilated to 8 mm using an angioplasty balloon (Mustang TM, PTA Balloon Catheter, Boston Scientific, Pulau Pinang, Malaysia) Fig. 3B. Final measurements revealed a portosystemic gradient of 8 mmHg.
12. To secure the percutaneous hepatic tract and reduce the risk of bleeding, a 8-mm Amplatzer vascular plug (Abbott Medical, MN USA) was deployed under fluoroscopic guidance Fig. 3C.
13. The final angiographic control confirmed the correct TIPS positioning Fig. 3D.

## Outcome

The patient remained hemodynamically stable throughout the procedure. Post-procedural Doppler ultrasound at 24 and 72 h confirmed TIPS patency with hepatofugal flow and decompressed varices. No bleeding, infection, or haemobilia were observed. The clinical course was favorable with stabilization of hemoglobin and no recurrence of bleeding (Tables 1 and 2).

**Table 1:** Comparison of alternative techniques for challenging TIPS

Technique	Main advantages	Limitations	Typical applications
CS	Combines real-time ultrasound and fluoroscopy; single transhepatic pass; percutaneous tract closure with vascular plug	Requires advanced expertise in hepatic ultrasound guidance	Salvage option after failed standard transjugular access
Gun-sight	Precise fluoroscopic alignment of snares; no need for advanced ultrasound	Requires dual vascular access, sometimes including splenic puncture	Complex anatomies, limited ultrasound visibility
DIPS	Direct IVC-to-portal vein tract; high technical success	Potential compromise of IVC; may affect transplant eligibility	Failure of standard TIPS, extensive portal vein thrombosis

**Table 2:** Step-by-step summary of the CS-TIPS procedure

Step	Description	Main devices
1	Transhepatic puncture of both hepatic and portal veins under ultrasound guidance with a single pass	5F Ring drainage needle
2	Confirmation of hepatic vein puncture with fluoroscopic overlap using a target catheter	5F Ring drainage needle
3	Establishment of through-and-through (“flossy”) access via snaring	20-mm Gooseneck snare
4	Tract dilation between hepatic and portal veins	5 × 40 mm PTA balloon
5	Deployment of covered stent graft between hepatic and portal veins	7 × 10 mm Viatorr stent graft
6	Post-dilatation of stent and gradient measurement	Mustang balloon catheter
7	Percutaneous tract closure to minimize bleeding risk	8-mm Amplatzer vascular plug

Table 2 summarizes the procedural steps of the CS technique, including the key tools required for each stage

## Discussion

TIPS has significantly evolved over the past two decades, gaining widespread acceptance in managing portal hypertension complications [1, 2, 8, 9]. Its main limitation remains the blind puncture of the portal vein following hepatic venous access [9]. Technical failure of the standard transjugular approach is not rare, particularly in patients with chronic liver disease or prior thrombosis [4, 5]. Exploring alternative techniques is essential, especially in complex anatomy or limited-resource settings. As recommended in the literature, a CE-CT scan is typically performed prior to the procedure to evaluate hepatic and portal venous anatomy and support planning in technically challenging cases [10, 11].

DIPS, which creates a tract between the IVC and portal vein via transhepatic puncture, is increasingly used as both a primary and salvage option. Despite its high success rate, it may hinder transplant strategies that preserve the native IVC. Intravascular ultrasound-guided TIPS improves accuracy and reduces needle passes and radiation exposure, though its availability is limited [12]. In this context, the CS technique should not be considered a competitor to DIPS, but rather a complementary option within the broader armamentarium for managing complex TIPS scenarios.

The gun-sight technique, using fluoroscopic alignment of snares in hepatic and portal veins, enables percutaneous needle passage through the liver. While it avoids expensive equipment and suits complex anatomy, the CS technique may offer a safer, less invasive alternative by eliminating splenic puncture and a second transhepatic access [13].

Percutaneous transhepatic portal vein puncture, though technically demanding, remains a viable option [6, 14, 15]. Most reports describe fluoroscopic guidance; real-time ultrasound is rarely emphasized. In our case, ultrasound allowed precise targeting of a recanalized portal branch, reducing off-target puncture and complications. Syed A. et al. described a similar single-needle technique, but our approach combines ultrasound and fluoroscopy, plus vascular plug deployment to secure the hepatic tract.

Correct needle placement in the hepatic vein was confirmed via cranio-caudal C-arm tilting, showing overlap between the Ring needle and catheter. Real-time ultrasound improves accuracy and reduces extrahepatic injury and trauma. Preliminary liver ultrasound is key to assess anatomy and identify an acoustic window aligning the right portal vein and hepatic vein for safe access [9]. These insights expand technical options for complex TIPS cases.

## Conclusion

The CS technique appears safer than DIPS, avoiding IVC puncture and its risks, and safer than the gun-sight method by eliminating splenic puncture, reducing access points, and enabling tract closure with a vascular plug. However, it should be reserved for operators with advanced expertise in ultrasound-guided hepatic procedures.

## Data Availability

Not applicable.
